# A data-driven SSM/PCA analysis approach for differential diagnosis of parkinsonism using ^11^C-PE2I PET

**DOI:** 10.1016/j.nicl.2026.103970

**Published:** 2026-02-18

**Authors:** Linus Falk, Carl Brunius, Tea Crnic Bojkovic, Lieuwe Appel, Charles Widström, Dag Nyholm, Torsten Danfors, My Jonasson, Mark Lubberink

**Affiliations:** a Molecular Imaging and Medical Physics, Department of Surgical Sciences, Uppsala University, Sweden; b Food and Nutrician Science, Department of Life Sciences, Chalmers University of Technology, Gothenburg, Sweden; c Medical Imaging Center, Uppsala University Hospital, Uppsala, Sweden; d Medical Physics, Uppsala University Hospital, Uppsala, Sweden; e Neurology, Department of Medical Sciences, Uppsala University, Sweden; f Neurology, Uppsala University Hospital, Uppsala, Sweden

**Keywords:** Parkinsonian syndromes, Differential diagnosis, Ensemble learning, ^11^C-PE2I PET, Dynamic brain scan, Cerebral blood flow, Dopamine transporter availability

## Abstract

**Background:**

Scaled Subprofile Modelling using principal component analysis (SSM/PCA) is a multivariate analysis technique primarily used in ^18^F-FDG PET brain studies to produce disease-specific patterns (DPs) and scalar scores aiding neurological diagnosis. SSM/PCA relies on well-characterized reference groups, posing challenges in real-world clinical datasets where diagnoses may be uncertain. A data-driven ensemble approach may offer a more robust alternative to random sampling when reference groups are unavailable.

**Objective:**

To apply SSM/PCA to dynamic ^11^C-PE2I-PET data for differential diagnosis of parkinsonism using a Monte Carlo cross-validation-inspired framework with ensemble prediction.

**Methods:**

Dopamine transporter availability, expressed as the specific binding ratio (SBR) relative to cerebellar gray matter and relative cerebral blood flow (R_1_) images from 47 healthy controls and 316 patients who underwent dynamic^11^C-PE2I-PET on a Discovery MI PET/CT scanner were included. Patients had a single most probable diagnosis of Parkinson’s disease (PD), dementia with Lewy bodies (DLB), or progressive supranuclear palsy (PSP) based on clinical information and the PET reading. A stratified 80/20 training/testing split was applied, repeated across 100 seeds, to generate DPs used for training ensemble classification models. Classification accuracy was assessed on the test-set.

**Results:**

Combining SBR and R_1_ improved accuracy yielding a balanced accuracy of 90%, with SBR primarily differentiating patients from healthy controls and R_1_ for differentiating between PD, DLB and PSP.

**Conclusions:**

Our results highlight the potential of an ensemble-based SSM/PCA method to assist differential diagnosis of parkinsonism. Future work will focus on including additional atypical parkinsonian disorders.

## Highlights

• Dynamic ^11^C-PE2I SSM/PCA using R_1_ and SBR for differential diagnosis of parkinsonism.
• Single-reference SSM/PCA can be unstable in clinical datasets with uncertain labels.
• Ensemble-SSM/PCA improves robustness through repeated reference sampling.
• High balanced accuracy achieved on an independent hold-out test set.

## Graphical abstract

**Figure:**
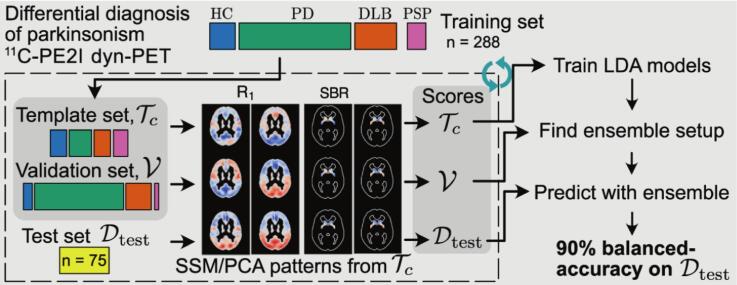

## 1 Introduction

Parkinsonian disorders are neurodegenerative chronic brain diseases, including idiopathic Parkinson’s disease (PD) and atypical parkinsonian disorders (APD) such as corticobasal degeneration (CBD), dementia with Lewy bodies (DLB), multiple system atrophy (MSA) and progressive supranuclear palsy (PSP). Typical clinical features of PD are associated with parkinsonism, i.e., tremor, rigidity, and bradykinesia, which is often present in patients with APD, making a differential diagnosis of these diseases challenging. The previously mentioned disorders affect the dopamine system and reduce dopamine transporter (DAT) availability. However, similar symptoms may also arise from vascular disease (vascular parkinsonism, VP), normal pressure hydrocephalus (NPH), frontotemporal dementia (FTD) or essential tremor (ET) which usually do not involve direct degeneration of the dopamine system (Cummings et al., 2011). Diagnosis based on clinical symptoms is difficult; in a longitudinal study of 232 patients with parkinsonism, the accuracy of clinical diagnosis was reported in the range from 65 to 93% (Adler et al., 2021). In a *meta*-analysis from 2016, movement disorder experts failed to accurately diagnose about 20% of PD patients (Rizzo et al., 2016).

Accurate early diagnoses are essential for several reasons. First, it enables more reliable prognostic predictions, as the disease course varies significantly between different parkinsonian disorders. Second, it ensures that patients receive the most appropriate symptomatic treatment, which can differ in effectiveness between those with PD and APD (Shih and Tarsy, 2007, Weaver et al., 2009). Moreover, accurate diagnoses are critical for clinical research, as including misdiagnosed patients in trials may obscure the true efficacy of disease-modifying therapies.

Molecular imaging methods, such as single-photon emission computed tomography (SPECT) and positron emission tomography (PET), can provide more precise diagnostic information (Brooks, 2010). In imaging-based differential diagnosis, DAT imaging can differentiate between diseases affecting the dopamine system and those that do not, while perfusion (^99m^Tc-HMPAO SPECT and glucose metabolism (^18^F-FDG PET) imaging can aid in determining differential diagnosis by identifying characteristic patterns of hypo- or hyperperfusion or metabolism in PD and APD. The combination of these two imaging techniques provides added diagnostic value (Du et al., 2024, Laere et al., 2006). However, it necessitates two separate patient visits and results in a relatively high cumulative radiation exposure. Recently, dual-phase imaging protocols have been proposed as an alternative. For example, early-phase ^18^F-FP-CIT PET, where a perfusion image is acquired during the first 10 min after injection, followed by a routine DAT image acquisition after 3 h, has demonstrated added value from a single radiotracer injection (Jin et al., 2017).

Dual-phase imaging reduces the number of visits but still obstructs smooth logistics and requires an extended stay for the patient at the hospital. A logistically easier and more patient-convenient alternative is a dynamic scan with the highly specific DAT tracer ^11^C-PE2I (Jonasson et al., 2013). Dynamic imaging of ^11^C-PE2I during 80 min, combined with tracer kinetic modeling, can be used to derive both the binding potential (BP_ND_), proportional to the concentration of available dopamine transporters, and the relative delivery (R_1_), representing cerebral blood flow ratio (CBFR) relative to cerebellar grey matter. In a comparative analysis, BP_ND_ and R_1_ from a single dynamic ^11^C-PE2I showed the same disease patterns as the commonly used dual-scan approach with ^123^I-FP-CIT SPECT and ^18^F-FDG PET (Appel et al., 2015). For a clinical implementation of ^11^C-PE2I we developed a dynamic scan protocol of 40 min, which still provides CBFR while BP_ND_ is approximated by the specific binding ratio (SBR) relative to cerebellar gray matter at 30–40 min after injection, causing only a minor decrease in accuracy compared to the 80 min protocol (Jonasson et al., 2017).

Several statistical approaches have been suggested to support early diagnosis based on ^18^F-FDG PET brain imaging, including covariance techniques such as principal component analysis (PCA) for biomarker identification (Moeller et al., 1987, Moeller and Strother, 1991). Scaled subprofile modelling principal component analysis (SSM/PCA) is a multivariate analysis technique commonly applied in ^18^F-FDG-PET studies to generate disease-specific patterns (DPs) and scores reflecting pattern expression. Reference patients and healthy controls are included to create DPs for prospective diagnosis. Alternatively, a conflicting disease group can be utilized as a reference group to generate a disease differential pattern (DDP). When using optimization methods such as masking or fine-tuning the selection of reference patients and controls, these methods can produce highly accurate diagnoses, and allow differentiating between HC, PD, MSA, and PSP with an up to 91% accuracy (Spetsieris et al., 2009). Automatic image-based classification with SSM/PCA has also been confirmed to agree well with autopsy, with an accuracy of 80%, identifying patients as PD or APD (Schindlbeck et al., 2021). Furthermore, the SSM/PCA method has previously been applied to dynamic ^11^C-PIB PET, showing that R_1_ and BP_ND_ parametric images can achieve classification performance comparable to FDG-SUVR and PIB SUV-R when distinguishing between healthy controls and patients with Alzheimer’s disease (AD) (Peretti et al., 2021).

However, real-world clinical datasets often contain uncertainty, and a single-model approach may be sensitive to sampling variation. Ensemble methods can address this by combining predictions from multiple classifiers (ensemble members) to produce a single, more reliable classification than any of the individual models typically can achieve. The most straightforward way to combine classifiers is by majority voting, where an unlabeled sample is assigned the class with the most votes. Furthermore, the number of ensemble members affects performance: too few risks underfitting, while too many may add computational cost without improving performance. The ensemble size can therefore be chosen in three different ways: 1) pre-selected by the user, 2) selected during training, for example, using out-of-bag estimation of the test error, or 3) post-selected by pruning the ensemble to a predetermined size (Rokach, 2009).

The aim of this proof-of-concept study was to retrospectively apply an SSM/PCA approach to dynamic ^11^C-PE2I-PET data for differential diagnosis of parkinsonism implementing a Monte Carlo cross-validation-inspired framework with ensemble prediction. Importantly, SSM/PCA performance relies on having a well-characterized reference group. However, this requirement becomes a limiting factor when working with real-world clinical datasets, where distinct groupings and diagnostic certainty may be lacking. In this study, we implement a Monte Carlo cross-validation-inspired framework with ensemble prediction to get around the limiting factors of SSM/PCA. Additionally, we combine DAT (SBR) and perfusion (R_1_) data into a single classification framework to improve diagnosis accuracy. By utilizing multiple reference groups and class-predicting models in an ensemble, we hypothesize that we reduce stochastic/random variability, improve the robustness, and emphasize the stable disease-relevant patterns from ^11^C-PE2I-PET SBR and R_1_ images.

## 2 Methods

### 2.1 Subjects

Healthy controls – Totally 47 healthy subjects were included from three different studies. Approvals were obtained from the Regional Board of Medical Ethics in Uppsala (diary numbers 2015–499 and 2017–219) or the Swedish Ethical Review Authority (EPM 2021–05230), and each subject signed an informed consent.

Patients – Approximately 1800 subjects were referred for assessment of Parkinsonian syndrome using ^11^C-PE2I-PET at Uppsala University Hospital between November 2016 and June 2024. The research presented in this paper was approved by the Swedish Ethical Review Authority (EPM 2020–01175), and informed consent was waived.

Inclusion criteria for DP and DDP generation were 1) a good quality Discovery MI ^11^C-PE2I PET scan without excessive motion and anatomical abnormalities, 2) a correct spatial normalization based on visual inspection, and 3) a single most likely diagnosis of PD, DLB, PSP, MSA-C, MSA-P, CBD, AD or FTD based on the SBR and R_1_ images (Eckert et al., 2005, Guedj et al., 2022, Morbelli et al., 2020) together with the referral information. Finally, patient groups should have a sufficient sample size after an 80/20 stratified train-test split (n≥20 in the training set), consistent with the minimum group size in the original groups used for pattern generation in the work of Spetsieris et al. (2009). Three patient groups comprising 316 patients fulfilled these methodological criteria, i.e., PD, DLB and PSP. A few demographic characteristics of the included subjects are presented in Table 1.

**Table 1:** Demography of subjects included in this study.

Diagnosis	n (M/F)	Age ± SD
Parkinson’s disease (PD)	122/83	69 ± 10
Dementia with Lewy bodies (DLB)	59/18	71 ± 9
Progressive supranuclear palsy (PSP)	19/15	73 ± 7
Healthy controls (HC)	21/26	62 ± 7

### 2.2 Data acquisition

All subjects underwent a 40 min dynamic ^11^C-PE2I PET scan starting simultaneously with controlled tracer bolus injection of 5 MBq/kg (5 mL at 1 mL/s followed by 35 mL saline at 2 mL/s) on a Discovery MI PET/CT scanner (GE Healthcare, Waukesha). Images were reconstructed into 14 frames (4x1, 2x2, 4x3, 4x5 min) using time-of-flight ordered subsets expectation maximization (TOF-OSEM) with 3 iterations and 34 subsets, point spread function recovery, and a 3 mm Gaussian post-filter.

### 2.3 PET data analysis

All PET data was analyzed using the in-house developed software PanDA (Perfusion and Dopamine transporter Availability). Inter-frame motion correction is performed by a rigid registration of each frame to the previously co-registered frame, using the 4th frame (3–4 min) as starting reference. Then, a 2–6 min summation image is spatially normalized to the MNI ICBM 2009c Nonlinear Symmetric template.

(https://www.bic.mni.mcgill.ca/ServicesAtlases/ICBM152NLin2009) using affine (linear) and B-Spline (non-linear) transformations. A volume of interest (VOI) atlas in template space, comprising 46 regions based on the CerebrA atlas (https://nist.mni.mcgill.ca/cerebra) (Manera et al., 2020), is incorporated in template space. This VOI atlas is transformed into the individual patient's space using the inverse of the transformation matrix and applied to the dynamic PET volume to extract a grey matter cerebellum time-activity curve, which serves as a reference.

Parametric SBR images were based on the 30–40 min interval with voxel-wise normalization of uptake values to cerebellar cortex, and expressed as the ratios minus one (Jonasson et al., 2017). Parametric R_1_ images were calculated using a voxel-wise basis function implementation of the simplified reference tissue model (SRTM) (Gunn et al., 1997, Lammertsma and Hume, 1996) with the cerebellar cortex as reference. The parametric images were then masked using the mean SBR or R_1_ images of the HC group. This process involved thresholding the right hemisphere (SBR >2.5 and R_1_ > 0.53), creating binary masks, and then mirroring the masks to create a symmetric striatum and grey matter mask for SBR and R_1_ images, respectively. SBR and R_1_ images were first flipped pairwise so that the most affected side (MAS), as defined from the SBR images, was on the right side. Subsequently, the R_1_ images were flipped such that the MAS defined by the R_1_ images was on the left. The SBR and R_1_ images were then additionally smoothed with a 6 mm full-width at half maximum (FWHM) Gaussian filter.

### 2.4 Stratified data split

A stratified 80/20 split was applied to divide the data into a training set $D_{train}$ and a hold-out test-set $D_{test}$. The hold-out test-set provides an unbiased estimate of the ensemble models’ performance on unseen data. Each sample consisted of a subject’s SBR image and R_1_ image, paired with a class label c∈C where the set of classes was defined as $C=\left\{HC,\;PD,DLB,\;PSP\right\}$.

### 2.5 Monte Carlo cross-validation

**Fig. 1:**
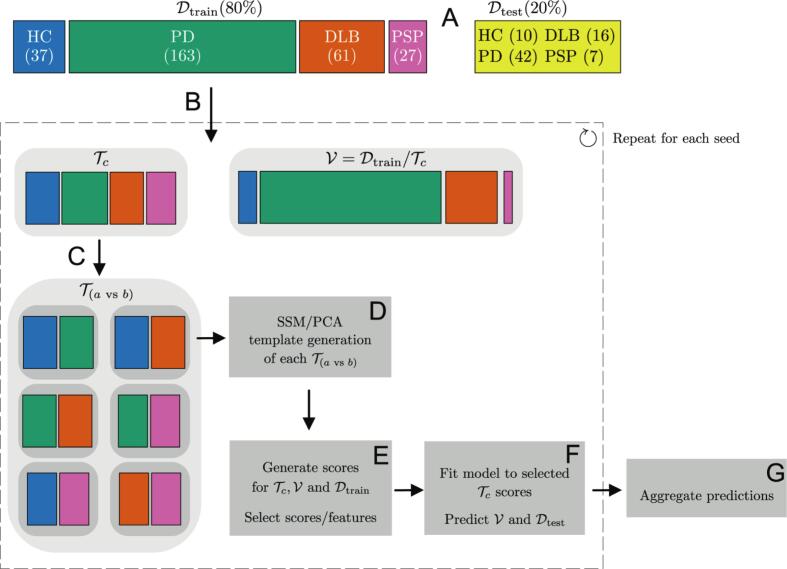
Pipeline for selecting subjects for pattern generation and validation. **A**: A stratified 80/20 split generated a training and a hold-out test-set. **B**: For each seed, sets of subjects from each class in the training set were randomly sampled to form the set $\;T_{c}$ for pattern generation. Unselected subjects for pattern generation served as a validation set V. **C**: Balanced pairwise sets ${(T}_{a\;vs\;b})$ were created from $T_{c}$ established for each class-pair combination by randomly downsampling the largest class to achieve equally sized pairs. **D:** SSM/PCA was applied separately to every balanced pairwise set ${(T}_{a\;vs\;b})$ and each parametric image type to generate DPs and DDPs. **E:** All subjects received a score for each of these DP and DDPs, which yielded in 12 candidate features. Each feature corresponded to one DP and DDP, providing a score for every subject. For each ${(T}_{a\;vs\;b})$, the candidate feature that yielded the greatest separation between two classes (Cohen’s d) was identified. **F:** A linear discriminant analysis (LDA) model was fitted to the features of the samples in $T_{c},$ and evaluated on the samples in V and $D_{test}$. **G:** The predictions of the validation and test-sets are then aggregated over each random seed for ensemble prediction.

A Monte Carlo cross-validation-inspired framework was established to select subjects for generating DPs and DDPs across 100 random seeds. For each seed, sets of subjects from each class $T_{c}$ were randomly sampled from $D_{train}$ for pattern generation. The number of subjects in each class was drawn from a discrete uniform distribution between 20 and 33, consistent with the minimum and maximum group sizes used for the original DP/DDPs generation in the work of Spetsieris et al. (2009). If the number of available subjects in a class was smaller than the randomly selected sample size, all but one subject were included from that class to allow for validation. For pattern generation, balanced pairwise sets $T_{\left(avs\;b\right)}$ were formed for each class-pair combination (a≠b) by randomly downsampling the largest class to achieve equal sample sizes. Unselected subjects for pattern generation served as a validation set V for each random seed. The pipeline for selecting subjects is illustrated in Fig. 1.

### 2.6 SSM/PCA analysis

Following the approach in the work by Spetsieris et al. (Spetsieris et al., 2013, 2009), the SSM/PCA method was implemented in Python. For each seed, SSM/PCA was applied separately to every balanced pairwise set $T_{\left(a\;vs\;\;b\right)}$ and each parametric image type. To reduce low values and noise in the R_1_ images, an individual mask was first created by applying a threshold of 35% of the maximum voxel value within the whole brain volume. For SBR, no threshold masking was used due to the characteristics of ^11^C-PE2I, where severely reduced or even absent DAT binding is part of the pathological pattern for both PD and some APD, making low values physiologically meaningful. The individual masks from all subjects in the balanced pairwise set were then multiplicatively combined to make one common mask that was applied to all subjects. Within each set, the SBR and R_1_ images of each subject were flattened to 1D arrays. The data was loaded into their respective data matrix $D_{R_{1}}$ and $D_{SBR}$, where each row corresponds to a subject and each column corresponds to a voxel. For each data matrix, an initial preprocessing step was performed to ensure that the PCA results would be invariant to subject and regional scaling effects. Specifically, the data matrix was log-transformed (with all values < $10^{-6}$, replaced by $10^{-6}$) and then row and column centered, resulting in a matrix of subject residual profiles (**SRP**) and a group mean profile (**GMP**) vector of the column means. Principal component analysis was then performed on the covariance matrix of the SRP, generating a score vector matrix **S**. Finally, the voxel vector PCA pattern matrix P was calculated as:

$$P=\left({SRP}^{T}\right)S,$$

where each column represents a principal component (PC) image pattern attributed to a percentage of the total variance, determined by the square root of the eigenvalues. Each of these PC patterns can be used to generate a score of pattern expression for a subject by calculating the subject’s subject residual profile ${SRP}_{s}$ vector with the GMP and then calculating the dot product with the PC pattern. Furthermore, any linear combination of PCs will also return a single score per subject. To construct a final DP/DDP $\left(P_{final}\right)$, the PCs accounting for the top 50% of the variance were linearly combined. Logistic regression, applied only to the scores of the subjects in $T_{\left(a\;vs\;\;b\right)}$, was used to determine the coefficients of this combination. The set of coefficients that minimized the Akaike information criterion (AIC) score was selected. Scores for all subjects were then calculated as:

$$score={SRP}_{s}∙P_{final}.$$

All scores were standardized to a Z-score using the mean and standard deviation of scores generated by subjects in class b in the $T_{\left(a\;vs\;b\right)}$ set. For visual examination of the generated DPs and DDPs can be reconstructed back into image format.

### 2.7 Feature selection

For each random seed, twelve DPs/DDPs were derived, and all subjects received a score for each of these patterns. This resulted in 12 candidate features, each corresponding to one DP/DDP and providing a score for each subject.

For feature selection, only the scores of subjects sampled for pattern generation ($T_{c}$) were considered. The class-pair combinations $T_{\left(a\;vs\;b\right)}$ (six in total) were evaluated in random order, where the candidate feature that yielded the greatest separation between the two classes, as measured by Cohen’s d, was identified. Once a feature was selected for a given class-pair, it was removed from the candidate pool to ensure a single use. This process yielded in one feature per class-pair combination and a feature vector f with six features per subject.

The selected features were used to train a linear discriminant analysis (LDA) model. LDA was chosen because it has been successfully used previously by Spetsieris et al. (2009), and requires no tuning of hyperparameters, reducing computational cost when repeated over multiple random seeds. The LDA model was fitted to features from subjects randomly sampled for pattern generation ($T_{c}$) and evaluated on the validation (V) and test ${(D}_{test})$ sets.

The added value of combining R_1_ and SBR-derived features was evaluated by comparing performance with models trained on features derived solely from R_1_ and SBR scores. In these cases, the feature vectors consisted solely of the six scores from DP/DDPs generated with the respective image type.

Additionally, we evaluated topographic similarity between SBR and R_1_ by correlating the validation-set expression scores for all derived DPs using Pearson’s correlation for each seed. Within each image type, topographic correlation was examined by correlating DP and DDP expression scores across the validation set for each seed.

### 2.8 Ensemble

The predictions on the validation sets were combined to estimate the optimal number of ensemble members (LDA models). Ensemble members were added sequentially in the order of their training. At each step, we combined their validation predictions using majority voting and recalculated balanced accuracy (defined as the average of sensitivity obtained across all classes), allowing us to track how performance evolved as the ensemble grew. To select the final ensemble configuration, we applied two different criteria. First, we identified the number of ensemble members M (with ≥5) that yielded the highest balanced accuracy. Second, we defined K as the smallest number of ensemble members (with K≥5) for which the change in balanced accuracy was less than 1% across a 20-seed window, indicating performance stabilization. Using the selected values M and K, majority voting was performed on the test-set predictions. Finally, the sensitivity, specificity, balanced accuracy, and accuracy on the test-set were assessed.

Since the reference diagnoses in this dataset were based on the SBR and R_1_ images together with referral information, an element of circularity is introduced. Consequently, performance metrics such as accuracy and balanced accuracy should be interpreted as measures of agreement with expert clinical classification rather than accuracy with respect to an independent diagnostic ground truth.

## 3 Results

### 3.1 SSM/PCA analysis

The mean explained variance in the derived PCA patterns for the SBR and R_1_ images is presented in Table 2, Table 3, respectively. For SBR, the first PC accounted, on average, for 50% or more of the explained variance. Except for the PD versus HC pattern, two PCs resulted in explained variances of at least 65% and, for combinations including PSP, about 80%. The standard deviations were relatively high in the first PC for all patterns.

**Table 2:** Mean and SD of the explained variance (%) in the first two PCs for the SBR data, accounting for at least 50% or more of the explained variance.

	PD vs HC		DLB vs HC		PSP vs HC		DLB vs PD		PSP vs PD		PSP vs DLB	
PC	Mean	SD	Mean	SD	Mean	SD	Mean	SD	Mean	SD	Mean	SD
1	0.46	0.06	0.52	0.08	0.70	0.08	0.55	0.09	0.69	0.07	0.71	0.05
2	0.20	0.05	0.23	0.04	0.12	0.04	0.14	0.03	0.11	0.03	0.12	0.02
Sum	0.66		0.75		0.82		0.69		0.80		0.83

**Table 3:** Mean and SD of the explained variance (%) with six PCs for the R_1_ data, accounting for at least 50% or more of the explained variance.

	PD vs HC		DLB vs HC		PSP vs HC		DLB vs PD		PSP vs PD		PSP vs DLB	
PC	Mean	SD	Mean	SD	Mean	SD	Mean	SD	Mean	SD	Mean	SD
1	0.17	0.02	0.29	0.02	0.22	0.01	0.20	0.02	0.15	0.02	0.23	0.02
2	0.11	0.01	0.09	0.01	0.10	0.01	0.11	0.01	0.10	0.01	0.11	0.01
3	0.07	0.01	0.07	0.01	0.07	0.00	0.08	0.01	0.08	0.01	0.08	0.01
4	0.06	0.00	0.05	0.01	0.06	0.00	0.06	0.01	0.07	0.01	0.06	0.00
5	0.05	0.00	0.04	0.00	0.05	0.00	0.05	0.00	0.05	0.00	0.05	0.00
6	0.04	0.00	0.03	0.00	0.04	0.00	0.04	0.00	0.05	0.00	0.04	0.00
Sum	0.50		0.58		0.52		0.53		0.49		0.56	

For R_1_, the first six PCs explained on average, 50–60% of the variance of the data over the 100 random seeds (Table 3). The highest average value was observed for the class-pair combination with DLB versus HC, whereas the lowest values were noticed for PD versus HC and PSP versus PD.

**Fig. 2:**
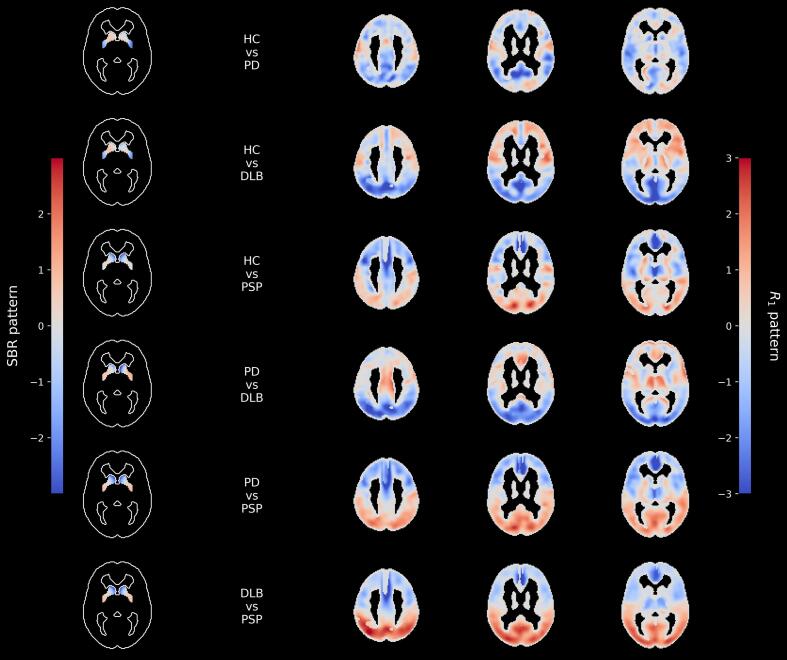
Average axial, Z-transformed SBR and R_1_ disease and disease differential patterns (DPs and DDPs) across 100 random seeds. The SBR DPs/DDPs patterns are characterized by changes along the posterior–anterior axis. The R_1_ DPs/DDPs are characterized by changes in perfusion, with a z score exceeding ±2 in the parietal, occipital, and frontal lobes.

Average Z-transformed DPs/DDPs, are shown in Fig. 2 for both SBR and R_1_. For SBR; changes extending along the striatal posterior–anterior axis were observed in all class-pair combinations. Distinct characteristics were found for mean R_1_ DPs/DDPs. Comparing the combinations DLB against PD or HC, the DP/DDP were characterized by strong changes in perfusion in the parietal and occipital lobes. On the other hand PSP against HC or PD showed a reduced perfusion in the frontal lobe.

**Fig. 3:**
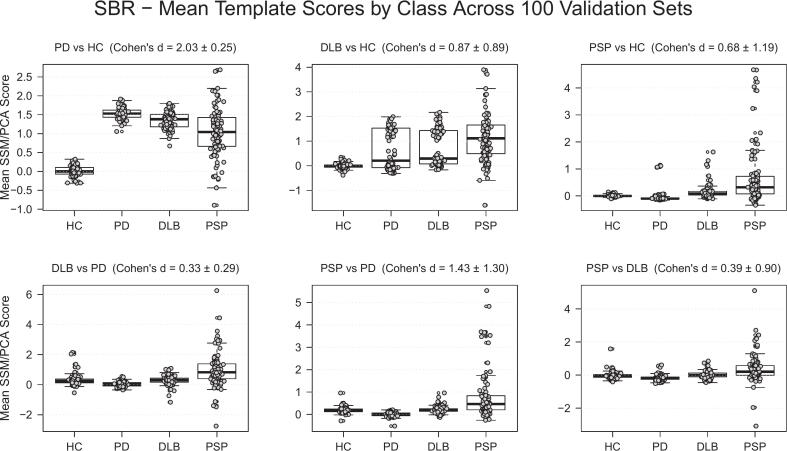
Distribution of the average score per class in each validation set, based on the output of each SBR DP/DDP over 100 random seeds.

**Fig. 4:**
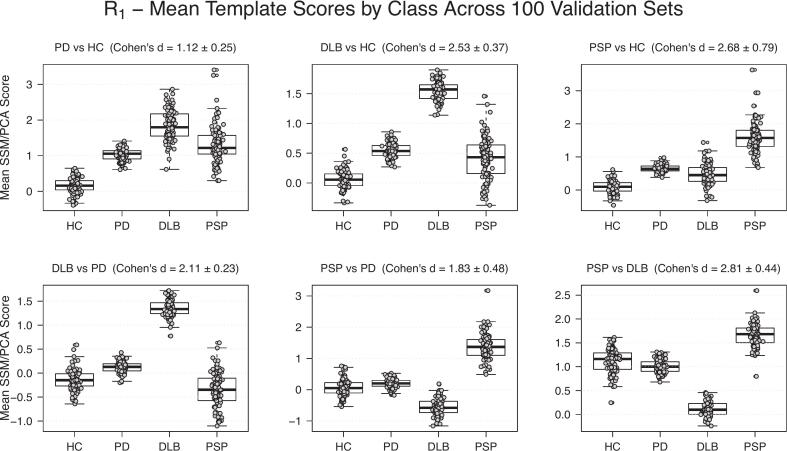
Distribution of the average score per class in each validation set, based on the output of each R_1_ DPs/DDP over 100 random seeds.

The average score per class was calculated across all validation sets for the SBR and R_1_ DPs/DDPs, in Fig. 3, Fig. 4, respectively. Cohen’s d for the class-pair combinations showed that the SBR pattern for the PD versus HC combination provided sharp separation of PD from healthy controls, but showed lower potential to separate the different diseases (Fig. 3). The scores of the R_1_ patterns showed fair discrimination overall, with a Cohen’s d ranging from 1.12 to 2.81, indicating a high ability to distinguish between PD, DLB, and PSP.

### 3.2 Ensemble evaluation

**Fig. 5:**
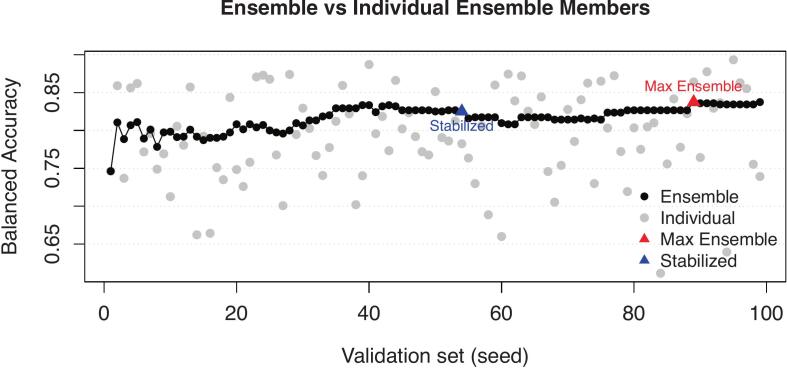
Balanced accuracy of all individual ensemble members on the seed-specific validation set and the incremental ensemble majority vote. The maximum ensemble balanced accuracy was achieved with 53 ensemble members. A stabilized point with less than 1% change over the last 20 steps was reached with 88 ensemble members.

The balanced accuracies of the ensemble and the individual ensemble members for the composite SBR + R_1_ ensemble are presented in Fig. 5. The maximum ensemble balanced accuracy for the SBR-only model was 59% (61% accuracy) with the minimum of 5 ensemble members; however, the model did not reach the predefined stabilization criterion. The R_1_-only model achieved a maximum ensemble balanced accuracy of 75% (65% accuracy) with 31 members and a stabilized balanced accuracy of 74% (64% accuracy) at 71 members. The composite SBR + R_1_ ensembles achieved the highest performance, with a maximum ensemble balanced accuracy of 84% (80% accuracy) at 89 members and a stable ensemble using 54 members with 83% balanced accuracy (79% accuracy).

The frequency of the selected features for all 100 ensemble members in the SBR + R_1_ ensemble is presented in Table 4. Of particular interest is the complete absence of the SBR DDPs scores for four combinations, as well as the low frequency of SBR features for DLB vs HC and R_1_ features for PD versus HC.

**Table 4:** Frequency of the selected features using a composite SBR + R_1_ ensemble model with 100 ensemble members.

$\left(T_{c_{1}}\;\mathrm{vs}\;T_{c_{2}}\right)$	SBR	R_1_
PD vs HC	79	26
DLB vs HC	22	82
PSP vs HC	0	96
DLB vs PD	0	99
PSP vs PD	0	99
PSP vs DLB	0	97

### 3.3 Classification results

The classification results on the hold-out test-set of the “Stabilized accuracy ensemble,” along with corresponding “Maximum Ensemble” results, are presented in Table 5. The SBR-only model did not reach the stability criterion, and therefore, only the “Maximum Ensemble” results are available. The composite SBR + R_1_ model outperformed the SBR-only and R_1_-only ensembles across most evaluation metrics.

**Table 5:** Classification results on the hold-out test-set of the “Stabilized accuracy ensemble” and “Maximum Ensemble” performance (in parentheses). Bold values represent the best-performing results for each metric.

	SBR-only		R_1_-only		Composite SBR + R_1_		
Class	Sensitivity	Specificity	Sensitivity	Specificity	Sensitivity	Specificity	Support (n)
HC	NA **(1.00)**	NA **(0.98)**	0.90 (0.90)	0.85 (0.85)	**1.00 (1.00)**	0.94 (0.94)	10
PD	NA (0.55)	NA (0.73)	0.57 (0.60)	0.94 **(0.97)**	**0.74** (0.71)	**0.97** (0.94)	42
DLB	NA (0.44)	NA (0.73)	0.94 (**1.00**)	**0.93 (0.93)**	**1.00** (0.94)	**0.93 (0.93)**	16
PSP	NA (0.14)	NA (0.88)	**1.00 (1.00)**	0.94 (**0.96**)	0.86 (0.86)	**0.96** (0.94)	7
**Accuracy**	NA (0.55)		0.73 (0.76)		**0.84** (0.81)		
**Balanced accuracy**	NA (0.53)		0.85 (0.87)		**0.90** (0.88)		

NA: not available.

## 4 Discussion

The aim of this study was to develop a framework to apply the SSM/PCA method to a clinical dataset of SBR and R_1_ images based on dynamic ^11^C-PE2I-PET data. We demonstrated that the presented SSM/PCA framework has the ability to discriminate between PD, DLB, PSP and healthy controls with a high accuracy. The dual-biomarker concept with the combination of SBR + R_1_ outperformed the classification performance using SBR and R_1_ separately across most evaluation metrics.

SSM/PCA has been previously applied to parametric ^11^C-PIB and ^18^F-FDG PET images in a study by Peretti et al., (Peretti et al., 2021) where the subjects were selected through a single instance of random sampling for template generation. Although a single instance of random sampling for pattern generation is straightforward, it does not account for the potential variability in single-instance random selection, which can be particularly problematic in a clinical dataset where disease progression is unknown and diagnostic certainty may be lacking. In our study, we repeated the process across multiple seeds, revealing substantial variability in the generated patterns, as demonstrated in the performance of the downstream classification on individual validation sets. Some variation in classification accuracy can be attributed to difficulties in classifying individual patients. The wide range of classification accuracy on the validation sets, ranging from 61% to 89% balanced accuracy, highlights the challenges of applying SSM/PCA to a clinical dataset.

The interpretation of the mean z-score SBR-based patterns requires some caution in contrast to the more intuitive understanding of ^18^F-FDG-based DPs where positive and negative voxel values often are described as “relative hyper or hypometabolism”. The derived SBR patterns contain both positive and negative voxel weights. These should not be interpreted as areas of increased DAT binding in PD, DLB, or PSP. Instead, positive voxels represent regions where these subjects show relative preservation compared to areas with the strongest degeneration. The SBR PD versus HC pattern showed the expected relative degeneration in the posterior striatum, whereas the PSP versus HC pattern showed more relative degeneration in the anterior striatum. This aligns with the known differences in striatal degeneration between PD and PSP, where more involvement of the anterior caudate has been reported in PSP than in PD (Oh et al., 2012). Similarly, the DLB versus HC pattern showed more signs of degeneration in the anterior striatal regions than the PD versus HC pattern, consistent with findings of lower caudate DAT binding in DLB compared to PD (Walker et al., 2004).

The derived mean z-score R_1_ patterns show similar characteristics as previously published ^18^F-FDG PET patterns, such as decreased metabolism in the parietal and occipital lobes in DLB and in the frontal lobes in PSP (Teune et al., 2010). These similarities support the relevance of the extracted perfusion patterns. However, the PD pattern in R_1_ was comparatively weak, aligning with the lower explained variance in the first principal component and the lower ensemble classification performance.

We observed a wide distribution of scores in the SBR patterns of HC versus DLB and HC versus PSP compared to those of the HC versus PD, as illustrated in Fig. 3. This could reflect the underlying pathological differences in striatal DAT degeneration in DLB and PSP compared to PD. A recent ^18^F-FE-PE2I PET study showed that DLB patients often exhibited a symmetric striatal DAT degeneration pattern compared to PD patients (Fedorova et al., 2023). This suggests the presence of subgroups within the dataset i.e., a mixture of subjects with mostly symmetrical and asymmetrical striatal degeneration. As a consequence, when sampling subjects randomly for SSM/PCA pattern generation, the relative frequency of asymmetric cases in the subsample will affect the resulting pattern, contributing to a broad distribution of scores. It is also possible that the lack of threshold masking is causing this wide distribution of scores with the lower signal-to-noise ratio in areas with a low binding ratio; however, this would not explain why the SBR PD versus HC scores are not showing a similar wide range.

Feature selection kept the features of patterns with the strongest class separation (Cohen’s d) to improve performance and reduce overfitting. While complete model-based selection (e.g., grid search over feature subsets) is possible, it is computationally intensive when repeated over many random seeds. Comparing the data-driven feature selection in this study with the clinical dual-scan approach reveals several similarities. In clinical practice, a DAT scan is typically used to identify degeneration of the dopamine system, while a follow-up ^18^F-FDG helps to differentiate PD from APD. Our results, both the Cohen’s d scores (Fig. 3) and the feature selection frequencies (Table 4), show a pattern that reflects this logic. The PD versus HC SBR feature was most frequently selected and effectively separated healthy controls from patients, while R_1_-derived features from disease versus disease comparison were selected most often, indicating that they contributed to a larger degree to the differentiation between parkinsonian disorders. Although the R_1_ patterns generally had higher discriminating capacity than SBR, the PD versus HC pattern was the least selected R_1_ pattern. This result agreed with the lowest Cohen’s d among the pairwise R_1_ classifications and the higher median score for the DLB and PSP classes on this pattern, indicating that this pattern is less discriminating than the DLB versus HC and PSP versus PSP patterns.

We further examined whether topographic similarity between SBR and R_1_ relates to these feature-selection trends by correlating the pattern expression scores of the validation sets. The PSP versus HC pair showed consistent coupling across SBR and R_1_, with significant correlation in 90 out of 100 seeds, whereas DLB versus HC and PD versus HC showed weaker correspondence (41 and 60 seeds, respectively). This is consistent with the SBR feature selection results in Table 4, where PSP versus HC is never chosen, suggesting reduced complementarity between SBR and R_1_ for this pair. Within the image type, topographic similarity was generally high across most pattern pairs (Supplementary Table 1), with more than 85 significant correlations for all DP and DDP pairs, except for the DLB versus HC and PSP versus HC comparisons, which were significant in 69 seeds. The high number of significant correlations across pairs and seeds suggests substantial similarities between these patterns in terms of topography.

Although each individual LDA classifier in the composite ensemble showed a wide range of balanced accuracy on the validation sets, by aggregating LDA predictions across different seeds and constructing an ensemble using either the stable or maximum criterion reduces variance in the final prediction, leading to more robust classifications. While other ensemble selection strategies exist, such as weighting methods that assign weights to ensemble members (either fixed or dynamically determined by performance) (Rokach, 2009), this simple order-agnostic approach aligns well with the explainability of the SSM/PCA method. Conceptually, repeatedly sampling the data to generate patterns resembles the bootstrapping techniques used to identify stable voxels in SSM/PCA pattern visualization (Habeck et al., 2008); however, here we average model classifications rather than voxel weights to produce robust classifications.

A recent replication, systematic review and *meta*-analysis of automated image-based parkinsonism diagnosis using ^18^F-FDG, with almost 500 subjects, reported a sensitivity of 84% and specificity of 94% for the classification of PD versus APD, using a two-step SSM/PCA classification framework in which PD versus APD is addressed in the first step and MSA versus PSP in the second step (Papathoma et al., 2022). Our SSM/PCA framework in a four-class setting, including the HC class, and not including MSA, represents a different diagnostic scenario in which non-PD/APD cases with similar symptoms may also be present. When using only the R_1_ image, which would be comparable to ^18^F-FDG, the sensitivity for identifying PD was 55% in our study. While this is lower than the binary classification sensitivity reported in the literature, this difference is expected because of the four-class setting we used. Furthermore, in the level-2 classification of the *meta*-analysis, the reported sensitivity and specificity for PSP versus MSA were 85% and 93%, respectively, while we cannot perform this exact comparison, our R_1_-only-based PSP classification achieved a sensitivity of 100% and a specificity of 96% within the multiclass setting considered here. In a more recent multicenter study with ^18^F-FDG, 73 PSP subjects representing three different phenotypes were classified using SSM/PCA with cross-validation against HC (n = 55) and PD (n = 58), yielding sensitivities of 80% and 80%, and specificities of 97% and 91%, respectively (Martí-Andrés et al., 2020). In addition, a comparison study investigated the added value of ^18^F-FDG-PET for distinguishing PSP (n = 41) versus HC (n = 46) subjects using SSM/PCA, they achieved a sensitivity in the range of 82–98% and a specificity of 70–92% (Buchert et al., 2024). Although the performance of our SSM/PCA framework is high on this dataset, caution is warranted when generalizing these findings. In particular, the limited number of PSP subjects and the use of an expert clinical agreement classification rather than an independent diagnostic ground truth may influence the apparent performance estimates.

The composite SBR and R_1_ ensemble improved PD sensitivity to 74%, which is substantially higher than using R_1_ alone and reflects the complementary information provided by combining perfusion and DAT availability. While still lower than the FDG-based sensitivity reported in the *meta*-analysis, the performance of the composite model's performance is consistent with the increased complexity of the multi-class setting and the inclusion of HC subjects. However, a comparison study using ^11^C-PIB R_1_ images as an alternative to ^18^F-FDG showed that R_1_ might be less sensitive to small changes (Peretti et al., 2019), suggesting potential limitations of using R_1_ as an alternative to ^18^F-FDG. Although R_1_ provides complementary information for differential diagnosis in the present study, it should not be interpreted as a definitive clinical replacement for FDG-PET. Perfusion and metabolism are often coupled, but neurodegenerative diseases may introduce partial decoupling (Iadecola, 2017).

The presented method makes efficient use of the parametric images from the 40-minute dynamic ^11^C-PE2I scan, which provides data to differentiate between healthy controls and multiple disease groups. The repeated ensemble method suppresses variability and increases robustness when applying the SSM/PCA method on a clinical dataset. At the same time, several limitations should be acknowledged. Firstly, the diagnosis of the PD, DLB and PSP subjects was based on clinical information at the time of scanning combined with the image read by an experienced nuclear medicine physician, without long-term follow-up. Hence some of the patients may be wrongly classified which limits the obtainable accuracy of the presented method. Furthermore, we cannot exclude the possibility that some components of the observed patterns may be influenced by disease stage and cognitive status within diagnostic categories. Such heterogeneity could affect the derived SSM/PCA patterns in addition to the effects of underlying atrophy and spatial normalization. Misalignment due to such structural differences may introduce artifacts or amplify regional differences unrelated to perfusion alone. Methodologically, this work was limited to LDA as the classifier. While LDA was selected for consistency with prior work, future studies should also explore alternative models.

Although the presented work is intended as a proof of concept, the proposed framework is designed for potential clinical translation. The data-driven SSM/PCA pipeline aims to reduce reliance on manual selection of well-characterized reference groups by providing a systematic, reproducible approach. Similar data-driven strategies could in the future be explored to improve labeling of large clinical datasets, further automating processes and potentially improving diagnostic accuracy through long-term follow-up. Finally, the method requires further validation before it can be considered for clinical applications.

## 5 Conclusion

By aggregating multiple reference groups of PD, DLB, PSP, and healthy controls using the SSM/PCA approach, we were able to distinguish between all conditions. The composite SBR + R_1_ data improved classification accuracy, reaching a balanced accuracy of 90%. The data-driven feature selection showed a pattern consistent with a ^123^I-FP-CIT SPECT + ^18^F-FDG clinical dual-scan approach, providing clinical grounding to this data-driven methodology. Repeating the procedure with a new random seed provides safeguards against stochastic/random variability and improves the robustness. These results highlight the potential of an ensemble-based SSM/PCA method to assist in differential diagnosis of parkinsonism. For a clinical implementation this SSM/PCA framework need to be further validated and extended to differential diagnoses beyond PD, DLB, and PSP, which is intended to be done in future work.

## Declaration of Generative AI and AI-assisted technologies in the writing process

During the preparation of this work the authors used Grammarly and ChatGPT in order to improve the flow and clarity of the text. After using this tool/service, the authors reviewed and edited the content as needed and take full responsibility for the content of the publication.

## CRediT authorship contribution statement

**Linus Falk:** Writing – original draft, Visualization, Methodology, Formal analysis, Conceptualization. **Carl Brunius:** Writing – review & editing, Supervision, Methodology. **Tea Crnic Bojkovic:** Writing – review & editing, Formal analysis, Data curation. **Lieuwe Appel:** Writing – review & editing, Project administration. **Charles Widström:** Software. **Dag Nyholm:** Writing – review & editing. **Torsten Danfors:** Writing – review & editing, Formal analysis, Data curation. **My Jonasson:** Writing – review & editing, Supervision, Data curation, Conceptualization. **Mark Lubberink:** Writing – review & editing, Supervision, Project administration, Funding acquisition, Data curation, Conceptualization.

## Declaration of competing interest

The authors declare that they have no known competing financial interests or personal relationships that could have appeared to influence the work reported in this paper.

## Data Availability

The dataset used in this study is available from the corresponding author upon reasonable request and ethics approval.
