# Supplementary material for: A data-driven SSM/PCA analysis approach for differential diagnosis of parkinsonism using 11C-PE2I PET
Source: Neuroimage Clin. 2026 Feb 18;49:103970. doi: 10.1016/j.nicl.2026.103970 (PMC12934278; doi:10.1016/j.nicl.2026.103970)
Supplement: Supplementary Data 1 [file mmc1.docx]

# Supplementary

Supplementary Figure 1: Flowchart of the patient selection procedure. PD, Parkinson’s disease; DLB, dementia with Lewy bodies; PSP, progressive supranuclear palsy.


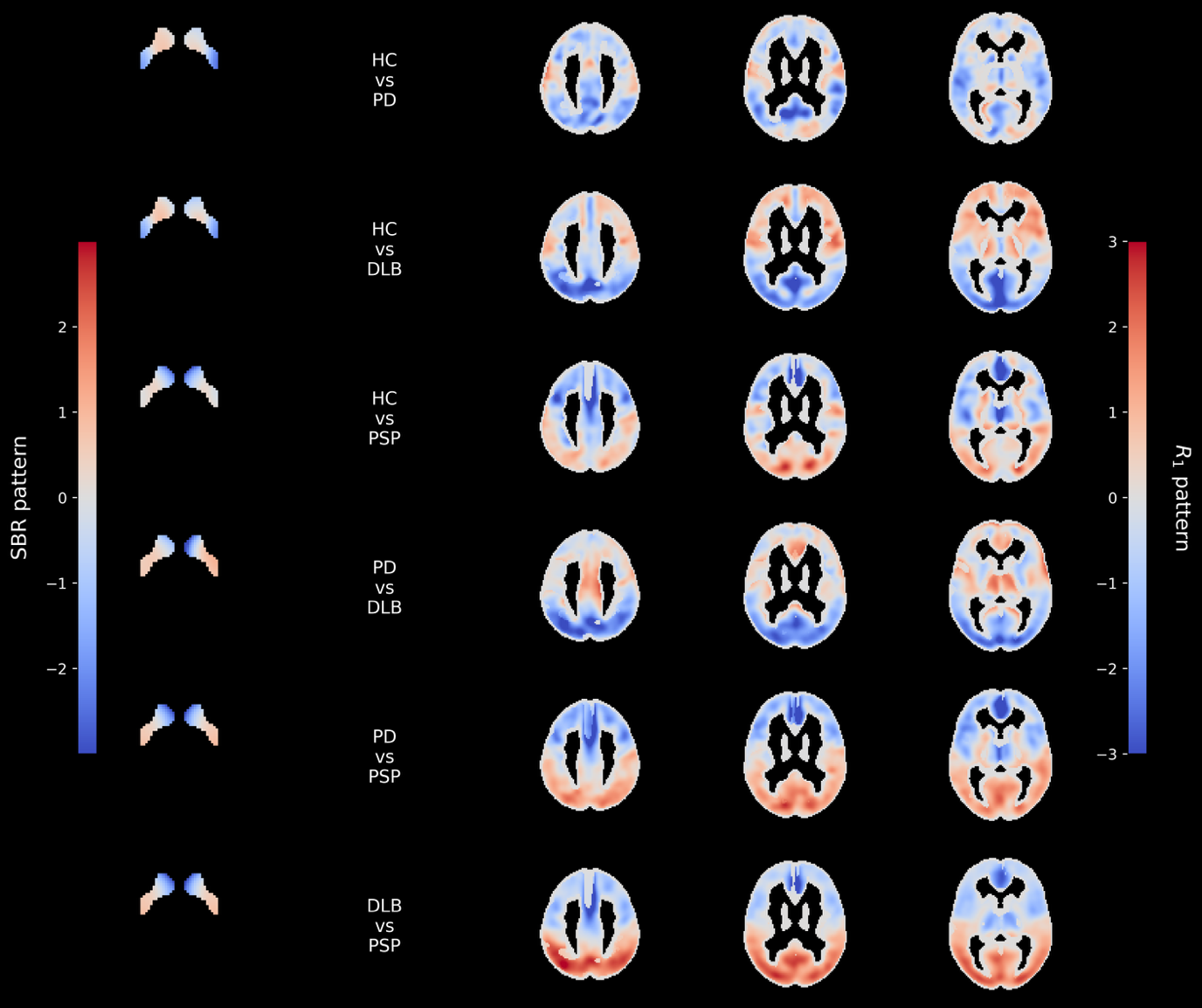


Supplementary Figure 2: Average axial, Z-transformed SBR and R_1_ disease and disease differential patterns (DPs and DDPs) across 100 random seeds. The SBR DPs/DDPs patterns are characterized by changes along the posterior–anterior axis. The R_1_ DPs/DDPs are characterized by changes in perfusion, with a z score exceeding $\pm2$ in the parietal, occipital, and frontal lobes.

Supplementary Table 1: Within-image-type topographic similarity of SSM/PCA-derived disease and disease-disease patterns, quantified by correlations of validation-set expression scores across 100 seeds.

|  | **Significant count**  **SBR** | **Significant count**  **R_1_** |
| --- | --- | --- |
| PD vs HC – DLB vs HC | 89 | 97 |
| PD vs HC – PSP vs HC | 86 | 98 |
| DLB vs HC – PSP vs HC | 94 | 69 |
| PD vs DLB – PD vs PSP | 99 | 91 |
| PD vs DLB – DLB vs PSP | 99 | 100 |
| PD vs PSP – DLB vs PSP | 100 | 100 |
